# 3D-equivariant graph neural networks for protein model quality assessment

**DOI:** 10.1093/bioinformatics/btad030

**Published:** 2023-01-13

**Authors:** Chen Chen, Xiao Chen, Alex Morehead, Tianqi Wu, Jianlin Cheng

**Affiliations:** Department of Electrical Engineering and Computer Science, University of Missouri, Columbia, MO 65211, USA; Department of Electrical Engineering and Computer Science, University of Missouri, Columbia, MO 65211, USA; Department of Electrical Engineering and Computer Science, University of Missouri, Columbia, MO 65211, USA; Department of Electrical Engineering and Computer Science, University of Missouri, Columbia, MO 65211, USA; Department of Electrical Engineering and Computer Science, University of Missouri, Columbia, MO 65211, USA

## Abstract

**Motivation:**

Quality assessment (QA) of predicted protein tertiary structure models plays an important role in ranking and using them. With the recent development of deep learning end-to-end protein structure prediction techniques for generating highly confident tertiary structures for most proteins, it is important to explore corresponding QA strategies to evaluate and select the structural models predicted by them since these models have better quality and different properties than the models predicted by traditional tertiary structure prediction methods.

**Results:**

We develop EnQA, a novel graph-based 3D-equivariant neural network method that is equivariant to rotation and translation of 3D objects to estimate the accuracy of protein structural models by leveraging the structural features acquired from the state-of-the-art tertiary structure prediction method—AlphaFold2. We train and test the method on both traditional model datasets (e.g. the datasets of the Critical Assessment of Techniques for Protein Structure Prediction) and a new dataset of high-quality structural models predicted only by AlphaFold2 for the proteins whose experimental structures were released recently. Our approach achieves state-of-the-art performance on protein structural models predicted by both traditional protein structure prediction methods and the latest end-to-end deep learning method—AlphaFold2. It performs even better than the model QA scores provided by AlphaFold2 itself. The results illustrate that the 3D-equivariant graph neural network is a promising approach to the evaluation of protein structural models. Integrating AlphaFold2 features with other complementary sequence and structural features is important for improving protein model QA.

**Availability and implementation:**

The source code is available at https://github.com/BioinfoMachineLearning/EnQA.

**Supplementary information:**

Supplementary data are available at *Bioinformatics* online.

## 1 Introduction

Predicting the structures of proteins from their sequences is crucial for understanding their roles in various biological processes. Various computational methods have been developed to predict protein structure from sequence information (Arnold *et al.*, 2006; Baek *et al.*, 2021; Hou *et al.*, 2019; Jumper *et al.*, 2021; Senior *et al.*, 2020; Xu, 2019; Yang *et al.*, 2020). However, some predicted structures are still far from the true structure, especially for some proteins lacking critical information such as homologous structural templates or residue–residue co-evolution information in their multiple sequence alignments. Besides, many computational methods produce multiple outputs for one input sequence. Thus, it is important to acquire a precise estimation of the model accuracy (EMA) for the predicted tertiary structurals, that is, their similarity or discrepancy with the native but unknown structure. Such estimation can help select the best models from the predicted candidates and identify erroneous regions in the models for further refinement.

Many methods for model quality assessment (QA) have been developed. For example, PCONS (Wallner *et al.*, 2007) and ModFOLDclustQ (McGuffin and Roche, 2010) use the comparison between 3D models to evaluate their quality. VoroMQA (Olechnovic and Venclovas, 2017) computes confidence scores based on the statistical potential of the frequencies of observed atom contacts. SBROD (Karasikov *et al.*, 2019) uses a smooth orientation-dependent scoring function with a ridge regression model. Deep learning-based QA methods have been reported. DeepQA (Cao *et al.*, 2016) uses a deep belief network and different agreement metrics. ProQ4 (Hurtado *et al.*, 2018) uses the partial entropy of the sequence characteristics with a Siamese network configuration. GraphQA (Baldassarre *et al.*, 2021) tackles the QA protein with graph convolutional networks based on geometric invariance modeling. Ornate (Pagès *et al.*, 2019) and DeepAccNet (Hiranuma *et al.*, 2021) are based on voxelized spatial information of the predicted models and 2D/3D convolution networks. DeepAccNet is one of the best-performing methods in the QA category of the Critical Assessment of Techniques for Protein Structure Prediction (CASP14) competition (Kwon *et al.*, 2021).

The pioneering development of the end-to-end deep learning method for protein structure prediction—AlphaFold2 (Jumper *et al.*, 2021) generated highly confident 3D structures for most protein targets in CASP14 as well as the recent release of a similar approach—RoseTTAFold (Baek *et al.*, 2021) presents notable improvements in structure prediction and brings new challenges for the model QA task because traditional QA methods developed for evaluating structural models predicted by traditional methods may not work well for the models predicted by the new methods such as AlphaFold2 (Kwon *et al.*, 2021). Since the software of the end-to-end approach, such as AlphaFold2 has been publicly released and is becoming the primary tool for tertiary structure prediction, it is important to develop corresponding QA methods to evaluate their models. Furthermore, since AlphaFold2 generates structural models with a self-reported per-residue local distance difference test (lDDT) (Mariani *et al.*, 2013) quality score, new QA methods should outperform (1) the consensus evaluation of a predicted model by comparing it with the reference models predicted by AlphaFold2 and (2) the self-reported per-residue lDDT score for models provided by AlphaFold2. And it would be interesting to investigate if and how various information extracted from AlphaFold2 predictions can be used to enhance the QA of 3D tertiary structural models. Finally, it is important to leverage the latest deep learning techniques of analyzing 3D objects.

The concept of rotation and translation equivariance in neural networks is useful for the analysis of rotation/translation-invariant properties of 2D and 3D objects in multiple domains, including 2D images (Cohen and Welling, 2016; Worrall *et al.*, 2017), quantum interactions (Schütt *et al.*, 2017) and 3D point clouds (Fuchs *et al.*, 2020; Satorras *et al.*, 2021; Thomas *et al.*, 2018). For equivariant networks, applying rotation and translation to the input results in a corresponding equivalent transformation to the output of the network. Invariance is a special case of equivariance, in which the same output is generated from the networks when the function returns scalar values such as distance or energy. Because the quality of a protein structural model is invariant to rotation and translation, it is desirable to use equivariant networks to predict model quality. As the locations of residues in a protein model can be represented as point clouds in 3D space, it is natural to represent a protein model as a graph, which can be equivariant to its rotation and translation. For example, the refinement step in RoseTTAFold (Baek *et al.*, 2021) uses an equivariant SE(3)-transformer architecture to update the 3D coordinates. GNNRefine uses a graph convolution network with invariant features for protein model refinement.

In this work, we present EnQA, a 3D equivariant graph network architecture for protein model QA. We evaluate the performance of our method on three different test datasets: the CASP14 stage2 models, the models of the Continuous Automated Model EvaluatiOn (CAMEO) and a collection of AlphaFold2 predictions for recently released protein structures in the Protein Data Bank (PDB). EnQA achieves state-of-the-art performance on all three datasets. It can distinguish the high-quality structural models from other models and performs better than the self-reported lDDT score from AlphaFold2. To the best of our knowledge, our method is the first 3D-equivariant network approach to the problem of model QA. It can effectively evaluate the quality of the models predicted by the current high-quality protein structure prediction methods such as AlphaFold2 that previous QA methods cannot.

## 2 Materials and methods

In this section, we first describe the training and test datasets and data processing procedure. Then, we define the input features to represent protein tertiary structures. Finally, we introduce the EnQA architecture and the implementation details.

### 2.1 Datasets

#### 2.1.1 CASP model QA dataset

We use structural models from server predictions for CASP8-14 protein targets (Stage 2 models if available) (Kwon *et al.*, 2021; Moult *et al.*, 1995) as one dataset, which can be downloaded from https://predictioncenter.org/download_area/. Models are first filtered by removing those with missing or inconsistent residues with respect to the corresponding experimental structure. The models from CASP8-12 are used for training. The models from CASP13 are used to validate the neural network and select its hyperparameters. The models from CASP14 are used as the benchmark/test dataset. The details of the data preparation are available in Supplementary Notes 1.1. As a result, there are 109 318 models of 477 CASP8-12 targets used for training, 12 118 models of 82 CASP13 targets used for validation and 9501 models of 64 CASP14 targets for the final benchmark/test, respectively. The models in the CASP dataset were generated by traditional protein structure prediction methods during the CASP experiments between 2008 and 2020. The average quality of the models is much lower than the models predicted by the state-of-the-art method—AlphaFold2.

#### 2.1.2 Alphafold2 model QA dataset

To create a QA dataset containing protein structural models predicted by the latest end-to-end prediction method—AlphaFold2, we first collect protein targets with sequence length ≥50 in the AlphaFoldDB Protein Structure Database (Tunyasuvunakool *et al.*, 2021) with corresponding experimental structures in PDB (https://www.rcsb.org/) (Berman *et al.*, 2000; Burley *et al.*, 2021) released after the cutoff date (April 30, 2018) of the structures on which AlphaFold2 was trained. In total, there are 4209 protein targets collected after filtering out identical ones. For each of these targets, we generate five structural models using AlphaFold2 with the model preset of ‘full_dbs’, restricting templates only to structures available before CASP14 (i.e. max_template_date = ‘2020-05-14’) to make sure the AlphaFold2 models of the targets are generated with only the information available before their experimental structures were released (see the details in Supplementary Note S1.2). The AlphaFold2 models of the targets are combined with the training dataset from CASP8-12 as a training data (CASP_AF_train). None of these targets in CASP_AF_train has above 30% sequence identity with any target in the CASP14 test/benchmark dataset consisting of CASP14 Stage 2 models (CASP14_test).

We also create another dataset that contains only protein structural models already available in the AlphaFoldDB with sequence length ≥50 and true structures available in PDB. In total, 6229 structural models for 6229 unique single-chain proteins with known Structural Class Of Proteins (SCOP) representative family domains (Andreeva *et al.*, 2014, 2020) are selected. The targets for testing are chosen from the data by two criteria: (1) their corresponding true structures were released after the start date of CASP14 (May 14, 2020) and (2) not sharing any SCOP representative family domains (Steinegger and Söding, 2017) with any target in the remaining data, resulting in 56 test targets in the test dataset (AlphaFold2_test). The remaining 6173 structural models are split into the training dataset (AlphaFold2_train) and validation dataset (AlphaFold2_val) according to the 80–20% ratio for training and optimizing the deep learning models. This data split strategy guarantees that the AlphaFold2_test dataset does not share the same protein family with the AlphaFold2 training dataset and validation dataset.

#### 2.1.3 CAMEO model QA dataset

To create an additional benchmark dataset, we use the recent models from CAMEO (Robin *et al.*, 2021). We download the protein structural models registered between September 4, 2021, to November 27, 2021, which include predictions from the latest predictors from different groups, such as RoseTTAFold (Baek *et al.*, 2021). Models are filtered by removing submissions containing only a partial sequence of the corresponding target. In total, 38 targets with 945 structural models are selected for benchmarking (CAMEO_test). The preprocessing procedure for the CAMEO dataset is described in Supplementary Note S1.2.

### 2.2 Features

We use a graph to represent a protein structural model, which contains node features and edge features. The node feature describes the information of each residue, while the edge feature describes the information for each pair of residues. We briefly describe each type of feature below.

#### 2.2.1 Node features

For an input protein chain with length *L*, the node features are created as follows. (1) One-hot encoding of amino acids (*L*, 20) is used. (2) Following the spherical convolutions on molecular graphs (Igashov *et al.*, 2021), we use the following three types of features to characterize the *geometric property* for each residue: the solvent-accessible surface area (*L*, 1), the size of Voronoi cell (*L*, 1) (Olechnovič and Venclovas, 2014) and the shortest topological distance to nearby solvent-accessible residues, which is also known as ‘buriedness’ (*L*, 1).

For models trained for CASP14 and CAMEO datasets, we leverage the information from AlphaFold2 predictions made for the protein sequence of each model to generate the quality features for the model. AlphaFold2 predictions used for feature generation are made with the template database curated before the release date of the experimental structure of any target in the PDB. The lDDT score of each residue computed with respect to an AlphaFold2 prediction for the same target (called a reference model) is used as a residue-level feature (*L*, 5). The AlphaFold2 self-reported lDDT score for each residue in the reference model is also used as a feature measuring the confidence of the reference model (*L*, 5). The final shape of the node features for each residue is (*L*, 33).

For the deep learning models trained and tested on the AlphaFold datasets (AlphaFold2_train and AlphaFold_test), no features from reference structures are used. Instead, we use the self-reported lDDT score (i.e. b-factor value) in the input PDB structural model generated by AlphaFold2 (*L*, 1). The final shape of the node features for each residue is (*L*, 24). Therefore, the deep learning models trained on AlphaFold data are a single-model QA method that only requires an input structural model as input to evaluate its quality.

#### 2.2.2 Graph edge features

For the deep learning models trained on the CASP_AF_train dataset, we first extract the logits from the distogram representation of the Alphafold2 predictions for a protein target, which represents the probability of the beta carbon (Cb) distance between two residues falling into pre-defined 64 distance bins, which has a shape (*L*, *L*, 64). From the 64-bin distogram, we then compute the probability of the distance error between two residues in a structural model falling into the nine distance bins defined by lDDT as follows:

(1) $${\;d}_{\mathrm{error}}^{i}=\frac{d_{\mathrm{upper}}^{i}+d_{\mathrm{lower}}^{i}}{2}-d_{\mathrm{model}},$$

(2) $$P^{n}=\sum_{i=1}^{64}P_{\mathrm{disto}}^{i}I_{d_{\mathrm{error}}^{i}\;\in {\mathrm{bin}}_{n}},$$

where $d_{\mathrm{error}}^{i}$ is the distance error (difference) between the AlphaFold2-predicted distance and an input model for the *i-*th distance bin of AlphaFold2 and $d_{\mathrm{upper}}^{i}$ and $d_{\mathrm{lower}}^{i}$ are the upper and lower bound of the *i-*th bin of the distogram, respectively. $d_{\mathrm{model}}$ is the distance between any two residues in the input model. $P^{n}$ is the probability of the distance error between two residues falling into the *n-*th distance bin defined by lDDT (Mariani *et al.*, 2013). $P_{\mathrm{disto}}^{i}$ is the softmax-normalized probability of the *i-*th distance bin from AlphaFold2 distogram. *I* is an indicator function which equals 1 if $d_{\mathrm{error}}^{i}$ falls into the range of the *n*-th bin defined by lDDT and 0 otherwise. The details of generating the pairwise distance error features of a model with respect to the distogram prediction of AlphaFold2 are available in Supplementary Note S2.1. Since we use five AlphaFold2 distogram predictions for each target and nine distance bins according to the definition of lDDT, this results in pairwise edge features with a shape (*L*, *L*, 45) for each pair of residues in a structural model. We also create contact probability maps by summing up all probabilities in AlphaFold2 distograms that fall into the bins with middle point ≤15 Å. The final binary contact map is the average from all five AlphaFold2 predictions to produce an additional edge feature with a shape (*L*, *L*, 1).

For the deep learning models trained on the AlphaFold2_train dataset, since no reference structural model is used, we do not include edge features from the agreement between the AlphaFold distograms and the model. Instead, we use the binary contact map computed from the input structural model with a cutoff of 15 Å. In addition, we use the representation from the transformer protein language models (Rives *et al.*, 2021) as protein sequence embedding. We choose the attention weights (*L*, *L*, 120) from the last layer of model ‘esm2_t6_8M_UR50D’ as the input feature.

#### 2.2.3 Spherical graph embedding edge features

**Fig. 1.**
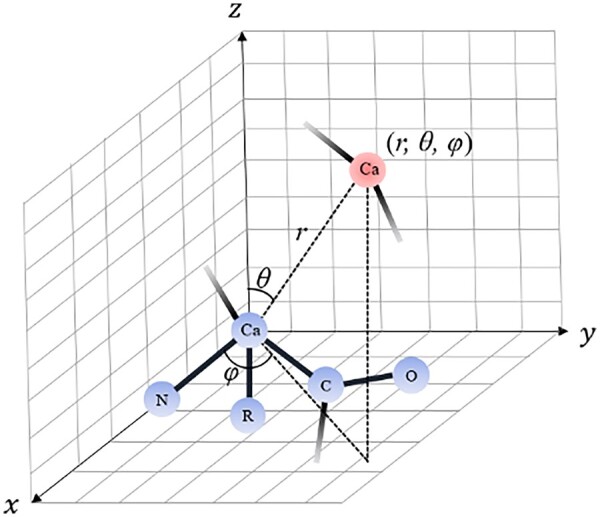
The illustration of the local spherical coordinate system. Different colors indicate atoms from different residues. Here, *θ, φ* and *r* are spherical angles and the radial distance for the vector between the alpha carbons (Ca) of two residues (blue and red)

We generate rotation-invariant graph embeddings following the Spherical Graph Convolutions Network (Igashov *et al.*, 2021) to use spatial information as spatial edge features. We first build the local coordinate frame for each residue in a structural model. We define the normalized Ca–N vector as the *x*-axis, the unit vector on the C–Ca–N plane and orthogonal to the Ca–N vector as the *y*-axis. The direction of the *y*-axis is determined by the one that has a positive dot product with the Ca–C vector. Naturally, the *z*-axis is the cross-product of *x* and *y*. We compute the spherical angles *θ* and *φ* of the vector between the Ca of each residue and that of any other residues with respect to this local spherical coordinate system. Figure 1 illustrates the local spherical coordinate system used in this work.

The spherical angles *θ* and *φ* are transformed into real spherical harmonics with the following formula:

(3) $$Y_{l}^{m}\left(\theta ,\;\varphi \right)=\;\sqrt{\frac{\left(2l+1\right)}{4\pi }\frac{\left(l-m\right)!}{\left(l+m\right)!}\;}P_{l}^{m}\left(\cos\theta \right)e^{im\varphi },$$

(4) Ylm=-2ImYlm if m<0Yl0            if m=02ReYlm if m>0.

Here, $Y_{l}^{m}\left(\theta ,\;\varphi \right)$: $S^{2}\to$ ℂ is a function defined on the surface of the unit sphere with degree *l* and order *m*, $Y_{l}^{m}$: ℝ → ℂ transform the complex spherical harmonics into their real forms. $P_{l}^{m}\left(\cos\theta \right)$ is the associated Legendre polynomials (Hobson, 1931). For spherical harmonics with degree *l*, there are 2*l *+ 1 orders in total. We choose spherical harmonics with degrees from 0 to 4 in the graph embeddings, resulting in 25 orders for each pair of spherical angles *θ* and *φ*. The final graph embeddings have shapes (*L*, *L*, 25) and are concatenated with the pairwise edge distance features as model input. The structural information of the protein models is incorporated while preserving the rotation/translation invariance property by using such embeddings from the local spherical coordinate frame.

### 2.3 3D-equivariant model architecture

**Fig. 2.**
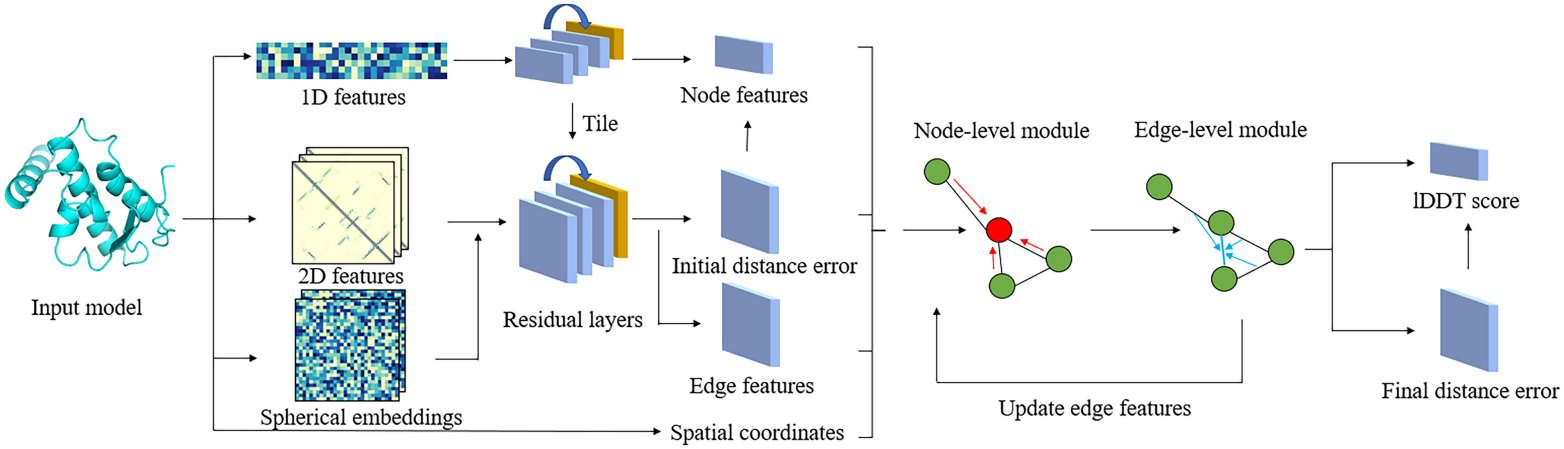
The illustration of the overall architecture of EnQA. The 1D/2D features from the input model are first converted into hidden node and edge features for the 3D-equivarant graph module. The spatial coordinates of Ca atoms of the residues are also used as an extra feature. The node and edge network modules update the graph features iteratively. In the end, the final per-residue lDDT score and distance errors of residue pairs are predicted from the updated node/edge features and spatial coordinates by the 3D-equivariant network

The overall architecture of our method is depicted in Figure 2. The processed 1D features (node features) are first processed with 1D convolutions to generate hidden node features. Then 2D features (both distance and graph embedding edge features) and the 2D tiling of the 1D hidden features are processed with a residual architecture with 5 blocks and 32 channels similar to the DeepAccNet (Hiranuma *et al.*, 2021). The goal is to predict an initial distance error as a classification task with nine bins. The distance error is converted into an initial quality estimation using the binary contact map described in Section 2.2.2. The equation for the *n*-th residue in input with length *L* is the following:

(5) $${\mathrm{score}}_{n}=\sum_{i=1}^{L}p_{ni}\left(\frac{p_{\mathrm{error}\leq 0.5Å}+p_{\mathrm{error}\leq 1Å}+p_{\mathrm{error}\leq 2Å}+p_{\mathrm{error}\leq 4Å}}{4}\right).$$

Here, $p_{ni}$ is the probability of the beta carbon distance between *n*-th and *i*-th residue in the binary contact map. $p_{\mathrm{error}}$ is the sum of the probability of the multi-class error prediction from the residual layers below different distance cutoffs. This score is combined with the other 1D node features as the node features for the following 3D-equivariant graph network. The 3D coordinates of Ca atom of each residue from the input model are updated by the graph network in a 3D-equivariant manner. The initial input coordinates and the updated coordinates are used to compute the final real valued distance error, which is used as an auxiliary output. The input graph for the 3D-equivariant graph network is constructed by connecting any residue pairs with distance ≤15 Å with an edge. The edge features for the graph network are the concatenation of the multi-class error prediction and a separate output of the residual layers for the pairs of the residues.

We use a variant of the *E*(*n*) Equivariant Graph Neural Networks (EGNN) (Satorras *et al.*, 2021) to process the node and edge features from the input graph and predict the final model quality score. Given a graph G=(V, E) with nodes $v_{i}\;\in \;V$ and edges $e_{ij}\in \;E$. Our 3D-equvariant network has a node-level module and an edge-level module. In the node-level module, the hidden node features $h_{i}\;\in \;R^{n}\;$and alpha carbon (Ca) coordinates $x_{i}\;\in \;R^{3}\;$associated with each of the residues are considered. The equation of the EGNN layers is the following:

(6) $${\;m}_{ij}=\varphi_{e}\left(h_{i}^{l},\;h_{j}^{l},\;{\left\|\left.x_{i}^{l}-x_{i}^{l}\right\|\right.}^{2},a_{ij}\right),$$

(7) $${\;x}_{i}^{l+1}=x_{i}^{l}+\frac{1}{N}\sum_{j\in N(i)}\left(x_{i}^{l}-x_{j}^{l}\right)\varphi_{x}\left(m_{ij}\right),$$

(8) $$m_{i}=\sum_{j\in N(i)}m_{ij}\;,$$

(9) $${\;h}_{i}^{l+1}=\varphi_{h}(h_{i}^{l},\;m_{i}).$$

Here, $h_{i}^{l}\;$and $h_{j}^{l}$ are the node features at layer *l*, $a_{ij}$ is the edge feature and $x_{i}^{l}$ and $x_{j}^{l}$ are the alpha carbon coordinates. $\varphi_{e},\;\varphi_{x}$ and $\varphi_{h}$ are multi-layer perceptron operations. $m_{ij}$ and $m_{i}$ are the intermediate messages for edges and nodes, respectively. The Ca coordinates are updated through each step so that its pairwise distance can reflect the distance map in the native PDB model and can be used to compute the final real value-based distance error when subtracting the distance map from the initial coordinates of the model.

For the edge-level EGNN module, inspired by the geometric transformer (Morehead *et al.*, 2021), we use edges in the original graph as nodes, and define the new node features as the original edge features. Unlike the edges in the node-level module, we use the *k*-nearest neighbors approach to define the edges in the edge-level module with *k* set to 3 to accommodate the memory limit for edge-level graphs. The coordinates of the edges are the midpoint of two ends and are always determined by node coordinates rather than updates from the edge-level module. Finally, we use the distances between the midpoints as the new edge attributes. The whole architecture can be trained end-to-end from the input features to the final lDDT score prediction. In addition to the EGNN-based graph layer, we also implemented a variant of the network by replacing the EGNN layers with a graph convolution network with kernels regularized by spherical harmonics functions as described in the SE(3)-transformer (Fuchs *et al.*, 2020) for comparison.

We use 6 Nvidia Tesla V100 32G GPUs on the Summit supercomputer and Horovod/Pytorch to train the method. The batch size is set to 1 for each GPU, resulting in an effective batch size of 6. We use the stochastic gradient descent optimizer with learning rate 1*e*−6, momentum 0.9 and weight decay 5*e*−5. We use the categorical cross-entropy as the loss function for initial distance error and the mean-squared error (MSE) loss for predicted lDDT scores as well as the final distance errors. The weight of the loss for predicted lDDT set to 5, while the weight of the other two errors is set to 1. We set the number of training epochs to 60 with early stopping when there are no improvements in validation loss for five consecutive epochs. Under our testing environment, the deep learning model can handle proteins with length up to 850 residues. Structural models with sequence length longer than 850 are cropped into segments of length up to 800 and the final results are rebuilt with the concatenation of all the segments.

## 3 Results

### 3.1 Model QA on the AlphaFold2 and other datasets

To compare the performance of EnQA with other state-of-the-art QA methods, we first evaluate it on generally high-quality AlphaFold2 structural models. We compare it with DeepAccNet (Hiranuma *et al.*, 2021), VoroMQA (Olechnovic and Venclovas, 2017) and ProQ4 (Hurtado *et al.*, 2018), which are all publicly available. We train EnQA, EnQA-SE(3) and EnQA-MSA that use the representations from transformer protein language models as extra features on AlphaFold2_train dataset without using reference structures to generate input features at all. Therefore, they are single-model QA methods. They are blindly evaluated on the AlphaFold2_test dataset (Table 1). The evaluation metrics used include residue and model-level MSE, mean absolute error (MAE) and Pearson correlation coefficient between the predicted lDDT scores and ground truth lDDT scores of the models. The average of the predicted per-residue lDDT scores for each model is calculated as the predicted global quality score of the model. The per-residue metrics are first computed for each model and are then averaged across all models. The self-reported lDDT scores of AlphaFold2 are used as the baseline method for comparison (named AF2-plddt).

**Table 1.** Results on AlphaFold2 test dataset (AlphaFold2_test)

Method	Per-residue			Per-model		
	MSE	MAE	Cor	MSE	MAE	Cor
AF2-plddt	0.0173	0.0888	0.6351	0.0105	0.0802	0.8376
DeepAccNet	0.0353	0.1359	0.3039	0.0249	0.1331	0.4966
VoroMQA	0.2031	0.4094	0.3566	0.1788	0.4071	0.3400
EnQA-MSA	**0.0090**	**0.0653**	**0.6778**	**0.0027**	**0.0386**	**0.9001**
EnQA	0.0093	0.0723	0.6691	0.0031	0.0462	0.8984
EnQA-SE(3)	0.0102	0.0708	0.6224	0.0034	0.0434	0.8926

Bold numbers denote the best results.

The results show that EnQA-MSA outperforms all other methods on all residue- and model-level metrics. For instance, the per-model correlation of EnQA-MSA is 0.9001, higher than 0.8376 of AF2-plddt, 0.4966 of DeepAccNet and 0.34 of VoroMQA. Compared with AF2-plddt, both EnQA-MSA and EnQA achieve significantly better per-residue and per-model MSE/MAE/correlation (*P *<* *0.01, paired *t*-test). The better performance than AF2-plddt shows that an independent QA method can evaluate AlphaFold2 models better than AlphaFold2’s built-in quality scores. All our three methods, including EnQA-SE(3) uses SE(3)-transformer architecture with the same features as EnQA-MSA, perform substantially better than the previous QA methods (DeepAccNet and VoroMQA) on this test dataset, clearly demonstrating the need of developing new QA methods for evaluating AlphaFold2 models.

In addition, we also evaluate all methods on CASP14_test dataset (Supplementary Table S1) and CAMEO_test dataset (Supplementary Table S2). For these datasets consisting of non-AF2 models, we additionally use five reference AlphaFold models predicted for each CASP14 target as reference to evaluate the CASP14 models. The average lDDT score between a CASP14 model and the five AlphaFold2 models is used as the predicted quality score of the model. This method is called AF2Consensus. Our method trained on the combination of CASP8-12 models and AlphaFold2 models (EnQA-Full) outperform all the other methods on both residue and model-level metrics, except its per-residue MAE and ranking loss of GDT-TS is slightly worse than AF2Consensus. Compared with AF2Consensus, EnQA achieves significantly better per-residue MSE/correlation, and per-model MSE/MAE, with *P *<* *0.01 (paired *t*-test), indicating the effectiveness of our method in scenarios with a wide range of model qualities.

### 3.2 Analysis of the performance on AlphaFold2-predicted models

**Fig. 3.**
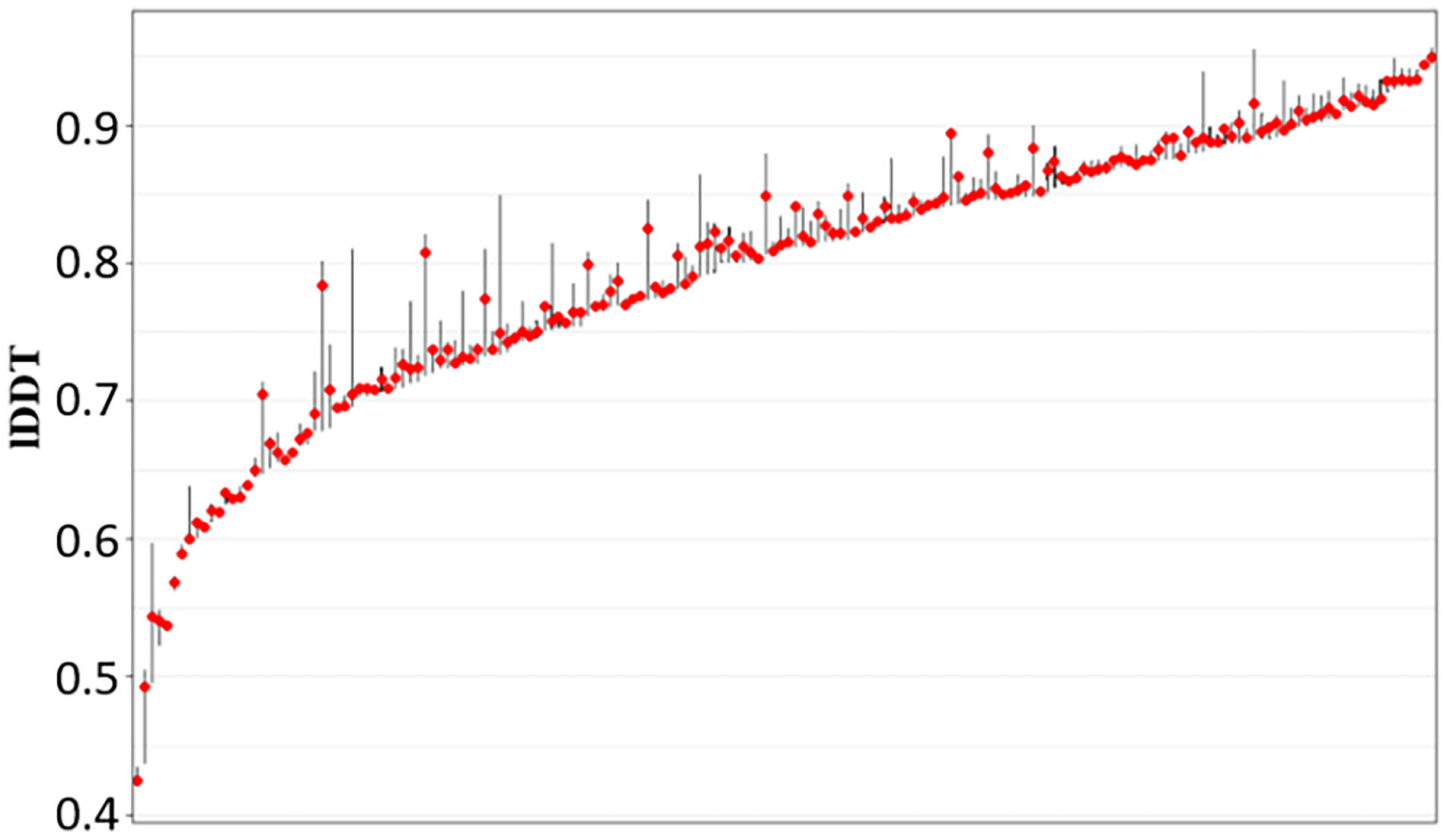
The distribution of lDDT scores of AlphaFold test models. The *x*-axis denotes the targets ordered by the mean lDDT of their models in increasing order. The red dots indicate the position of the median and the bars indicate the upper and lower ranges of model quality of each target

We first examine the distribution of model quality of the models in the AlphaFold2_test dataset (Fig. 3). The average true lDDT score for all models is 0.8034, with 79.82% above 0.7. The distribution of model quality of the CASP and CAMEO datasets is provided in Supplementary Figures S1 and S2. The results indicate that the structure models in the AlphaFold2 test dataset have much higher average quality than the CASP and CAMEO test datasets.

**Fig. 4.**
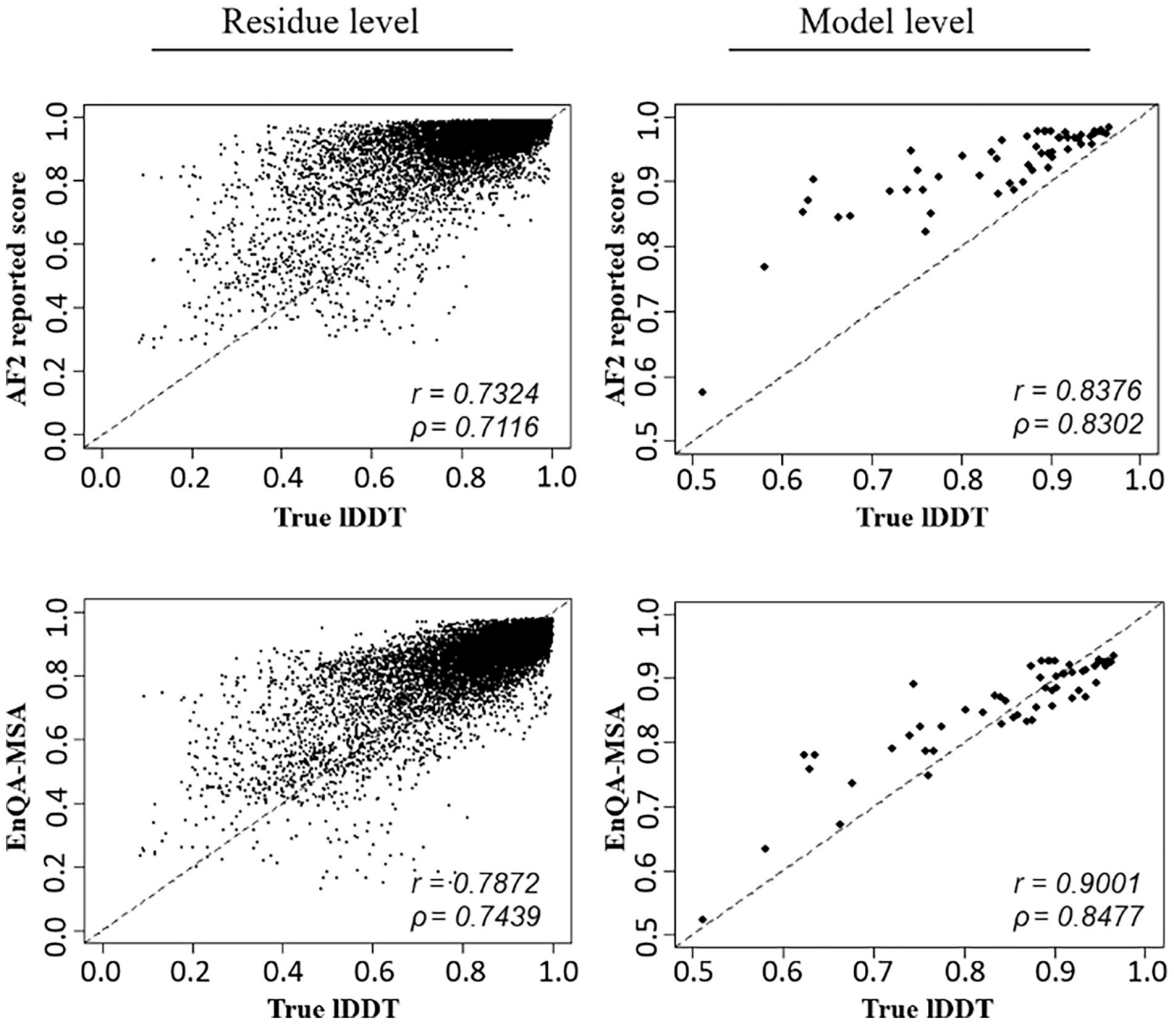
The comparison between the predicted and true lDDT scores for AlphaFold2_test models for the two methods (AF2 reported score and EnQA-MSA). The residue-level correlation is computed for all residues at once, which is different from the average of the residue-level correlation in each model (used in Sections 3.1 and 3.2). *r*, Pearson correlation coefficient; *ρ*, Spearman correlation coefficient. The lDDT scores predicted by EnQA-MSA have higher correlation with the true lDDT scores than AlphaFold2 self-reported scores

**Fig. 5.**
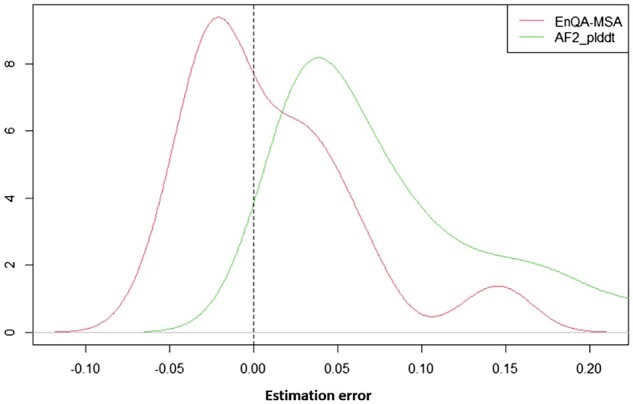
The distribution of estimation error between the predicted and true lDDT scores on AlphaFold2_test dataset. The difference between AF2_plddt scores and true pLDDT scores (green) is significant (*P *<* *0.01), but the difference between pLDDT scores predicted by EnQA-MSA and true pLDDT scores (red) is not significant (*P* = 0.117)

We further investigate the characteristics of the predictions of EnQA-MSA and the AlphaFold2 self-reported lDDT score on the AlphaFold2_test dataset (Fig. 4). The predicted scores of EnQA-MSA have a higher correlation with the true lDDT scores than AlphaFold2 self-reported quality scores. At both the residue and model level, the AlphaFold2 self-reported score tends to systematically overestimate the quality of the models, but EnQA-MSA substantially reduces the overestimation bias (Fig. 5). There is a significant difference between the true lDDT scores and AF2 reported scores (*P *<* *0.01, paired Wilcoxon signed-rank test), but there is no significant difference between EnQA predictions and the true lDDT scores (*P *=* *0.3545, paired Wilcoxon signed-rank test). This may partially explain why an independent QA method like EnQA-MSA can evaluate AlphaFold2-predicted structures better than AlphaFold2’s quality scores. It is also worth noting that the overestimation by AlphaFold2’s self-reported pLDDT score may not always be an error since there is also some error in experimental structures and AlphaFold2-predicted structures may be more accurate than them in some cases.

### 3.3 Analysis of the impact of features

**Fig. 6.**
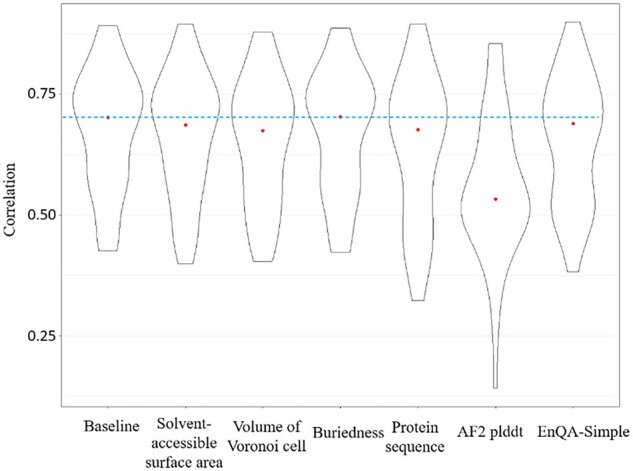
The comparison of residue-level Pearson’s correlation coefficient when different features are randomly permuted for model QA. The red dots indicate the position of the median

We examine the impact of different input features on the prediction performance of our QA model. We calculate the residue-level Pearson’s correlation coefficient between predicted lDDT scores and true lDDT scores on the AlphaFold2_test dataset (Fig. 5). We use EnQA-MSA as the baseline model and report the prediction performance when each type of feature (sequence, solvent-accessible surface area, volume of Voronoi cell, buriedness and AlphaFold2 self-reported score) is excluded during model training. A larger change in Pearson correlation coefficient indicates a higher impact. The detailed metrics of all models used in feature importance analysis are listed in Supplementary Table S3. The analysis shows that the AlphaFold2 self-reported confidence score (AF2 plddt) is the most important feature as its exclusion causes the largest drop in the Pearson’s correlation coefficient (*P *<* *0.01, paired *t*-test). However, the performance of the deep learning model without using the confidence score from AlphaFold2 still outperforms the other QA methods (DeepAccNet and of VoroMQA) by a large margin, indicating the effectiveness of the model architecture. Excluding sequence information also results in a significant decrease in the model performance (*P *<* *0.01, paired *t*-test). The results also show that one hand-crafted feature (buriedness) has almost no impact on the prediction accuracy, while the other two hand-crafted features (the solvent-accessible surface area and the volume of Voronoi cell) have some minor impact. Thus, we experiment with a simplified model without these three features, which yields slightly lower performance than EnQA-MSA (Fig. 6). The best performance of EnQA-MSA using multiple features demonstrates the importance of integrating multiple complementary features to improve the prediction performance, which also partially explain why it performs better than AlphaFold2 self-reported confidence score.

## 4 Conclusion

In this article, we introduce EnQA, a novel 3D-equivariant network method for protein QA. Our approach utilizes both the geometric structural features of an input model and the features extracted from AlphaFold2 predictions. The network is developed as an equivariant framework with the node and edge features passing through the node and edge-level graph networks. Performed computational experiments on diverse structural model datasets prove that EnQA achieves the state-of-the-art performance of protein QA. More precisely, on both CASP14 and recent CAMEO protein structures, EnQA outperforms all other methods on most evaluation metrics, including using AlphaFold2 predictions as reference to evaluate models. Furthermore, our method performs better than the self-reported lDDT score of AlphaFold2 in evaluating high-quality AlphaFold2 models. On all the test datasets, EnQA performs substantially better than the previous QA methods, demonstrating the value of using 3D-equivarnant architecture and AlphaFold2-based features. Also, we show that the input features extracted from structural models have a complementary effect with the information extracted from AlphaFold2 predictions, especially for those models on which EnQA performs better.

The huge success of AlphaFold2 and its self-reported quality score in protein structure modeling raised the question of the usefulness of EMA methods (Kwon *et al.*, 2021). However, even with AlphaFold2, in many cases, there are still predicted structures far from the true structures (Chakravarty and Porter, 2022), especially when there is no critical information such as good multiple sequence alignments or homologous structural templates available. The results in this work show that there is still room of improvement for evaluating AlphaFold2-predicted structures. There is a need to develop EMA methods to effectively rank AlphaFold2 models or to identify the potential regions of the models with low quality. As AlphaFold2 has become the standard tool for protein structure prediction, the next-generation EMA methods should focus mostly on AlphaFold2-predicted structures that have much higher average quality than structures predicted by traditional protein structure prediction methods. Therefore, the training and test data for the new EMA methods need to evolve accordingly as shown in this work.

To the best of our knowledge, our method is the first 3D-equivariant network approach to leveraging information from AlphaFold2 predictions to improve model QA. It may be further expanded for other 3D protein structure prediction tasks such as protein structure refinement and quaternary structure evaluation by using task-specific training datasets.

## Supplementary Material

btad030_Supplementary_Data

## Data Availability

The source code and datasets of this project are available at https://github.com/BioinfoMachineLearning/EnQA.
