# Supplementary material for: 3D-equivariant graph neural networks for protein model quality assessment
Source: Bioinformatics. 2023 Jan 13;39(1):btad030. doi: 10.1093/bioinformatics/btad030 (PMC10089647; doi:10.1093/bioinformatics/btad030)
Supplement: btad030_Supplementary_Data [file btad030_supplementary_data.docx]

Supplementary Notes

# 1 Selection of Datasets

## 1.1 CASP14 benchmark dataset protocol

CASP14 target list from the QA results (68 in total)

<https://predictioncenter.org/casp14/targetlist.cgi?view=regular&assis_type=all&view=all&field=t.release_date&order=ASC>

Remove 6 targets ('T1048', 'T1072s1', 'T1062', 'T1070', 'T1080', 'T1077'

) that are not evaluated in global QA benchmark as is described in the official assessment (66 remaining) <https://onlinelibrary.wiley.com/doi/full/10.1002/prot.26192>

Remove two targets without publicly available native structure (64 remaining)

"T1085" "T1086"

"T1098 MESHI_SERVER_TS4" is excluded due to incomplete prediction compared to the reference structure (486/538 residues).

## 1.2 Targets from CAMEO dataset

We first filter the models by removing those predictions with inconsistent sequences with the corresponding reference structure. Targets are excluded for further analysis if less than three models are left for them after filtering.

# 2 Feature generation

## 2.1 Distance error

The segregation of the lDDT bins is defined as [-∞, -4.0, -2.0, -1.0, -0.5, 0.5, 1.0, 2.0, 4.0, +∞]

The segregation of AlphaFold distogram bins is defined as [2.000, 2.3125 , 2.625 , 2.9375 , 3.25 , 3.5625 , 3.875 , 4.1875 , 4.5 , 4.8125 , 5.125 , 5.4375 , 5.75 ,6.0625 , 6.375 , 6.6875 , 7. , 7.3125 , 7.625 , 7.9375 , 8.25 , 8.5625 , 8.875 , 9.1875 , 9.5 , 9.812499, 10.125 , 10.4375 , 10.75 , 11.0625 , 11.375 , 11.6875 , 12. , 12.3125 , 12.625 , 12.9375 , 13.25 , 13.5625 , 13.875 , 14.1875 , 14.5 , 14.8125 , 15.125 , 15.4375 , 15.75 , 16.0625 , 16.375 , 16.6875 , 17.000 , 17.312498, 17.625 , 17.9375 , 18.25 , 18.5625 , 18.875 , 19.1875 , 19.5 , 19.8125 , 20.125 , 20.4375 , 20.75 , 21.0625 , 21.375 , 21.6875, 22.000 ]

For any residue pair of distance $d_{model}$ in the model, we define the distance error between the predicted Alphafold model and input model for the *i-th* distance bin of Alphafold as:

$$d_{error}^{i}= (d_{upper}^{i}+d_{lower}^{i})/2-d_{model}$$

Here $d_{upper}^{i}$ and $d_{lower}^{i}$ are the upper and lower bound of the *i-th* bin of the distogram.

We then compute the probability of the distance error between two residues falling into the *n-th* distance bin defined by lDDT as:

$$P^{n}=\sum_{i=1}^{64} P_{disto}^{i}I_{d_{error}^{i} \in{bin}_{n}}$$

Here $P_{disto}^{i}$ is the Softmax-normalized probability of the i*-th* distance bin from Alphafold distogram. $I_{d_{error}^{i} \in{bin}_{n}}$is an indicator function which equals 1 if $d_{error}^{i}$ falls into the *n-th* bin defined by lDDT and 0 otherwise. In practice, we set the first segregation of AlphaFold bins to 0.

**Figure S1.** The distribution of lDDT score in the benchmark dataset for CASP14_test models. The targets are ordered by mean lDDT. The red dots indicate the position of the median.


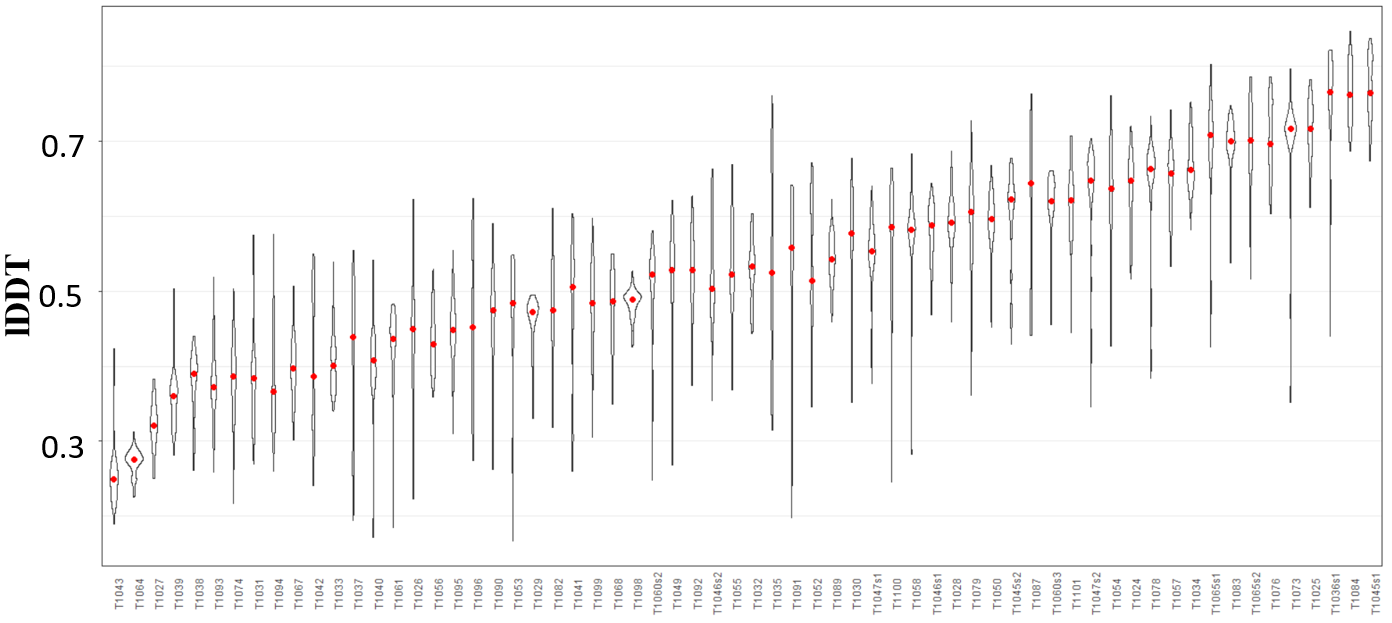


**Figure S2.** The distribution of lDDT score in the benchmark dataset for CAMEO_test models. The targets are ordered by mean lDDT. The red dots indicate the position of the median.


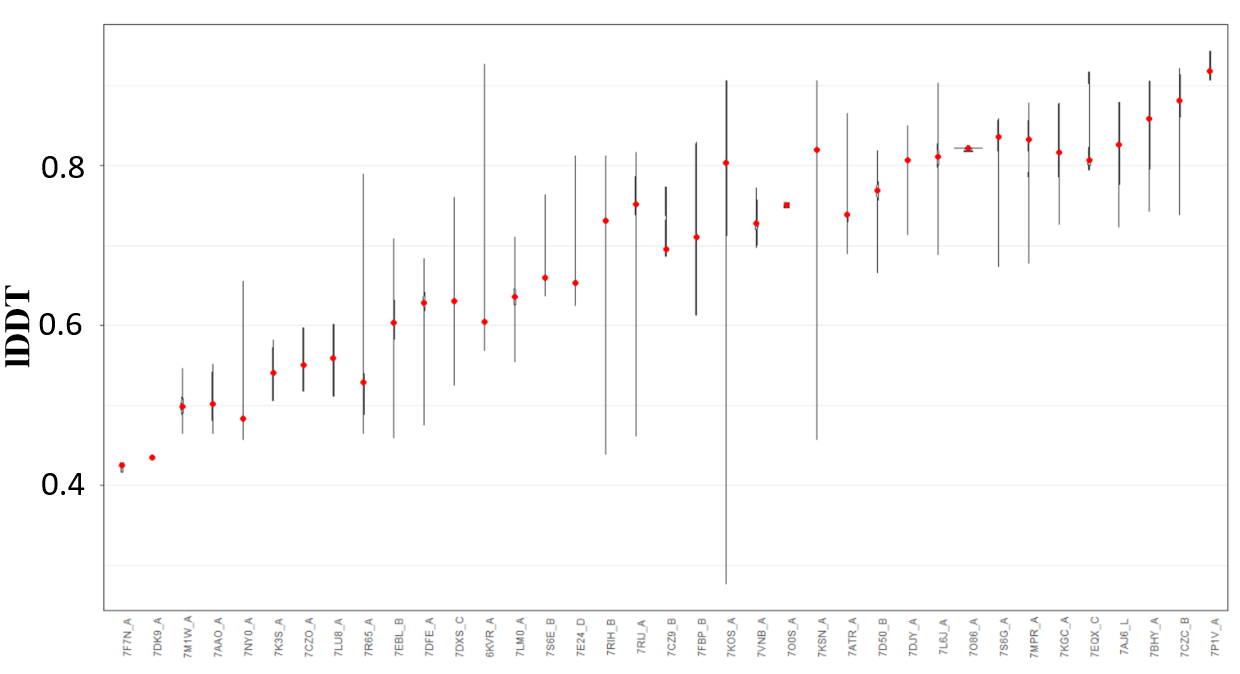


**Table S1**. The QA results on the CASP14 test dataset (CASP14_test). Bold denotes the best result.

| Method | Per-residue | | | Per-model | | | Ranking loss | |
| --- | --- | --- | --- | --- | --- | --- | --- | --- |
|  | MSE MAE Cor | | | MSE MAE Cor | | | lDDT GDT-TS | |
| AF2Consensus | 0.0057 | **0.0439** | 0.8596 | 0.0018 | 0.0244 | 0.9612 | 0.0092 | **0.0328** |
| DeepAccNet | 0.0254 | 0.1249 | 0.5725 | 0.0137 | 0.0945 | 0.7459 | 0.0444 | 0.0933 |
| VoroMQA | 0.0686 | 0.2115 | 0.3929 | 0.0466 | 0.1840 | 0.4620 | 0.0614 | 0.1175 |
| ProQ4 | 0.0296 | 0.1331 | 0.4493 | 0.0113 | 0.0806 | 0.7292 | 0.0570 | 0.1021 |
| EnQA-Full | **0.0049** | 0.0451 | **0.8676** | **0.0015** | **0.0227** | **0.9648** | **0.0088** | 0.0331 |
| EnQA-SE(3) | 0.0070 | 0.0607 | 0.7903 | **0.0015** | 0.0228 | 0.9611 | 0.0116 | 0.0323 |

**Table S2** The QA results on the CAMEO test dataset (CAMEO_test).

| Method | Per-residue | | | Per-model | | | Ranking loss | |
| --- | --- | --- | --- | --- | --- | --- | --- | --- |
|  | MSE MAE Cor | | | MSE MAE Cor | | | lDDT GDT-TS | |
| AF2Consensus | 0.0084 | 0.0535 | 0.8529 | 0.0036 | 0.0353 | 0.9191 | **0.0054** | **0.0105** |
| DeepAccNet | 0.0245 | 0.1215 | 0.6636 | 0.0146 | 0.1006 | 0.7250 | 0.0144 | 0.0193 |
| VoroMQA | 0.1297 | 0.3175 | 0.4561 | 0.1094 | 0.3125 | 0.5512 | 0.0470 | 0.0537 |
| ProQ4 | 0.0684 | 0.2163 | 0.4498 | 0.0508 | 0.1961 | 0.5374 | 0.0656 | 0.0673 |
| EnQA-Full | **0.0061** | **0.0508** | **0.8602** | **0.0017** | **0.0272** | **0.9517** | 0.0068 | 0.0132 |
| EnQA-SE(3) | 0.0085 | 0.0681 | 0.7764 | 0.0021 | 0.0340 | 0.9335 | 0.0115 | 0.0190 |

**Table S3.** Metrics for quality assessment of AlphaFold2_test model dataset of models used in the feature important analysis.

| **Method** | **Per-residue** | | | **Per-model** | | |
| --- | --- | --- | --- | --- | --- | --- |
|  | **MSE MAE Cor** | | | **MSE MAE Cor** | | |
| plddt | 0.0148 | 0.0870 | 0.5541 | 0.0064 | 0.0617 | 0.7094 |
| No - Surface | 0.0090 | 0.0652 | 0.6657 | 0.0026 | 0.0374 | 0.9008 |
| No - Volume | 0.0096 | 0.0688 | 0.6536 | 0.0032 | 0.0435 | 0.8948 |
| No - Buriedness | 0.0091 | 0.0664 | 0.6717 | 0.0028 | 0.0403 | 0.8815 |
| No Sequence | 0.0094 | 0.0672 | 0.6380 | 0.0029 | 0.0393 | 0.8903 |
| EnQA-Simple | 0.0091 | 0.0668 | 0.6538 | 0.0026 | 0.0391 | 0.8977 |
